# Ternary Gas Mixture Quantification Using Field Asymmetric Ion Mobility Spectrometry (FAIMS)

**DOI:** 10.3390/s19133007

**Published:** 2019-07-08

**Authors:** Yasufumi Yokoshiki, Takamichi Nakamoto

**Affiliations:** 1 Department of Information and Communications Engineering, School of Engineering, Tokyo Institute of Technology, Kanagawa 226-8503, Japan; 2 Laboratory for Future Interdisciplinary Research of Science and Technology, Institute of Innovative Research, Tokyo Institute of Technology, Kanagawa 226-8503, Japan

**Keywords:** quantification of gas mixtures, FAIMS, odor, e-nose

## Abstract

Gas mixture quantification is essential for the recording and reproducing odors, because an odor consists of multiple chemical compounds. Gas mixture quantification using field asymmetric ion mobility spectrometry (FAIMS) was studied. Acetone, ethanol, and diethyl ether were selected as components of a ternary gas mixture sample as representatives of the ketone, alcohol, and ether chemical classes, respectively. One hundred and twenty-five points with different concentrations were measured. The results were evaluated by error hypersurface, variance, and the coefficient of variation. The error hypersurface showed that it is possible to reach the target composition by following the error-hypersurface gradient. Successful convergence was achieved with the gradient descent method in a simulation based on the measurement data. This result verified the feasibility of the quantification of a gas mixture using FAIMS.

## 1. Introduction

Gas mixture quantification is an essential technology for odor recording [1], since an odor consists of multiple chemical compounds. Odor recorders and reproducers could be applied to various technical fields [2], if such devices were realized. For example, they could be employed in teleolfaction [3], odors for virtual reality [4], etc. Gas chromatography–mass spectrometry (GC–MS) is generally used for gas quantification, since it can separate gases easily and detect low concentrations of gas. However, it is expensive and does not work in real time. Moreover, the mixture composition of odor components should be quantified in an odor recorder. Several methods of gas mixture quantification using a sensor array such as the quartz crystal microbalance (QCM) method have been proposed [2]. It becomes difficult to determine the concentration of each gas because the QCM sensor array has a collinearity problem when the number of components in a gas mixture increases.

Ion mobility spectrometry (IMS) is an ion separation and detection method [5]. It is used for drug [6] and explosive detection [7], due to its high sensitivity. Meanwhile, field asymmetric ion mobility spectrometry (FAIMS) [8] is especially promising because it provides plenty of information. FAIMS uses an asymmetric field for ion separation using mobility which is dependent on the electric field [9].

FAIMS has been applied in various research fields [5], e.g., vegetable disease detection. Rutolo et al. realized potato storage disease detection using gas analysis [10]. Sinha et al. reported real-time detection for storage infections in stored potatoes and onions [11]. FAIMS has also been used for medical diagnostics [12]. Covington et al. reported the detection of a patient at risk of gastrointestinal toxicity during pelvic radiotherapy [13]. Sahota et al. achieved tuberculosis detection using human breath [14]. Arasaradnam et al. reported that the noninvasive diagnosis of pancreatic cancer could be achieved through the detection of volatile organic compounds in urine [15]. Plat et al. reported the noninvasive detection of anatomic leakage by urinary analysis [16]. FAIMS has also been used in other fields. Pfammatter et al. reported that FAIMS improved the accuracy and dynamic range of quantitative proteomic analyses [17]. Kontunen et al. reported tissue identification with surgical smoke analysis [18]. Sutinen et al. reported that the identification of breast tumors from diathermy smoke [19]. 

FAIMS has been used for odor analysis; for example, Surakka et al. reported a system prototype employing IMS to record the odor of jasmine oil [20]. Li et al. reported that FAIMS could be used for the odor assessment of interior automobile components [21]. However, gas mixture quantification using FAIMS has not yet been conducted, as previous researchers focused only on odor classification. Gas mixture quantification using FAIMS is the focus in this study. A ternary gas mixture was measured using FAIMS, and the feasibility of this method to quantify gas mixtures was examined.

## 2. Materials and Methods

**Figure 1:**
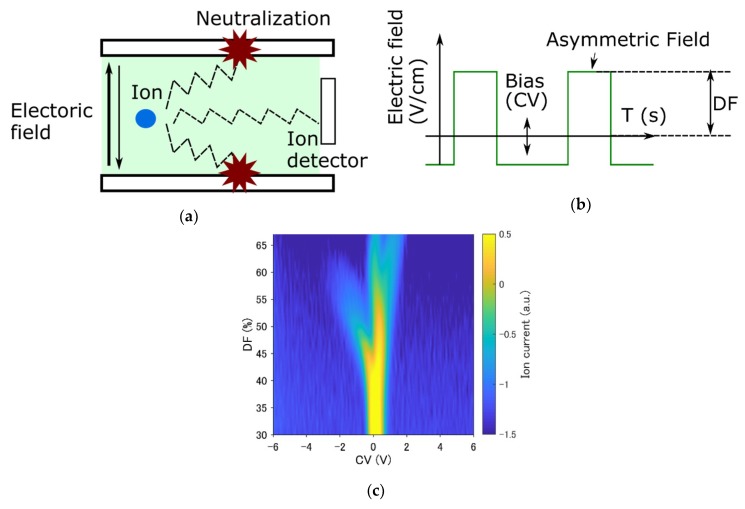
The mechanism of field asymmetric ion mobility spectrometry (FAIMS). (**a**) Ion movement in the electrodes. (**b**) Details about the asymmetric field. (**c**) An example of FAIMS response.

The FAIMS mechanism is shown in Figure 1a. The analyte was ionized with a Ni-63 ionizer. Ions were transported thorough the electrodes. Ions are affected by the electric field force, and their movement is accelerated in the up-and-down direction when an asymmetric field is applied. The electric field applied to electrodes had an asymmetric shape, as shown in Figure 1b. The maximum peak is called the dispersion field (DF), and the bias voltage is called the compensation voltage (CV). The CV and DF are swept, and the ions reach the ion current detector if the displacement is balanced. The ion current (IC) as a function of the CV and DF is the response of FAIMS, as shown in Figure 1c.

### 2.1. FAIMS

OLP-EK-023 (AtonARP, Tokyo, Japan) was used as a FAIMS apparatus. It used FAIMS CORE (Owlstone, Cambridge, UK). The range of the CV was between −6 V and 6 V. The DF could reach up to 250 V in the prototype [22]. The frequency of the asymmetric voltage was 26 MHz. The gap width between electrodes was 35 μm, and the gap length was 300 μm.

### 2.2. Measurement System

**Figure 2:**
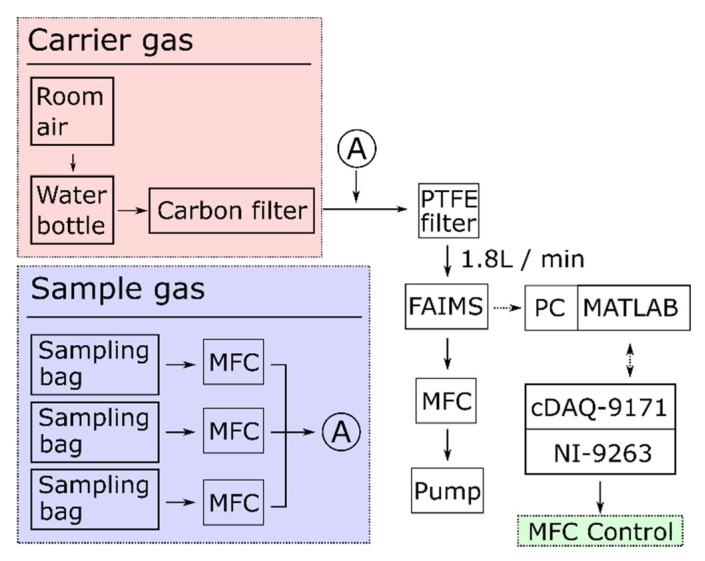
Block diagram of the measurement system.

A block diagram of the measurement system is shown in Figure 2. Ambient air through a carbon filter was used for the carrier gas. A water bottle was used to bubble the carrier gas to avoid low humidity. Sample gases were packed into a fluorine-containing resin bag (sampling bag) (GL Science, Tokyo, Japan). A polytetrafluoroethylene (PTFE) filter (T300A025A, Advantec, Tokyo, Japan) was used for removing pollutants. The gas in the sampling bag was measured by a photo ionization detector (PID) (ppbRAE 3000, RAE Systems, Sunnyvale, CA, USA), and then it was supplied to FAIMS. Mass flow controllers (MFCs) (MF-C series, HORIBA STEC, JAPAN) controlled the flow rates of the sample gases. Because the flow rate was fixed at 1.8 L/min in the FAIMS apparatus, regulated by another MFC, the concentration of each gas C (ppm) at the corresponding MFC can be calculated by:

(1) $$C=v\;\cdot C_{\mathrm{bag}}/1.8,$$

where C_bag_ is the gas concentration in the sampling bag and v is the flow rate (L/min) of the MFC. A digital to analog converter (DAC) module (cDAQ-9171 and NI-9263, NI, US) controlled the MFCs in MATLAB in order to collect data automatically.

### 2.3. Measurement Method

Acetone, ethanol, and diethyl ether were selected for components of the ternary gas mixture sample, being representatives of the ketone, alcohol, and ether chemical classes respectively. FAIMS ionization strongly depends on proton affinity [23]. Compounds with different functional groups were selected since proton affinity is influenced by functional group [24]. Moreover, these compounds are easy to handle.

Liquid samples were injected into sampling bags with a syringe. A compressor generated the air, which was dried by an air dryer (CF3-02, IAC, Kanagawa, Japan) and cleaned by a carbon filter before being packed into each sampling bag. Five different flow rates (0.0, 0.05, 0.1, 0.15, 0.20, and 0.25 L/min) were selected. Then, 125 mixture compositions of ternary gas mixture were measured. The DF range was set from 30% to 67.9%; 19 points were measured. The CV range was set from –6 to 6 V (512 data points). If a low-concentration gas was measured after a high-concentration gas analysis, the measurement was stopped for 30 seconds to clean the FAIMS apparatus.

### 2.4. Data Evaluation

**Figure 3:**
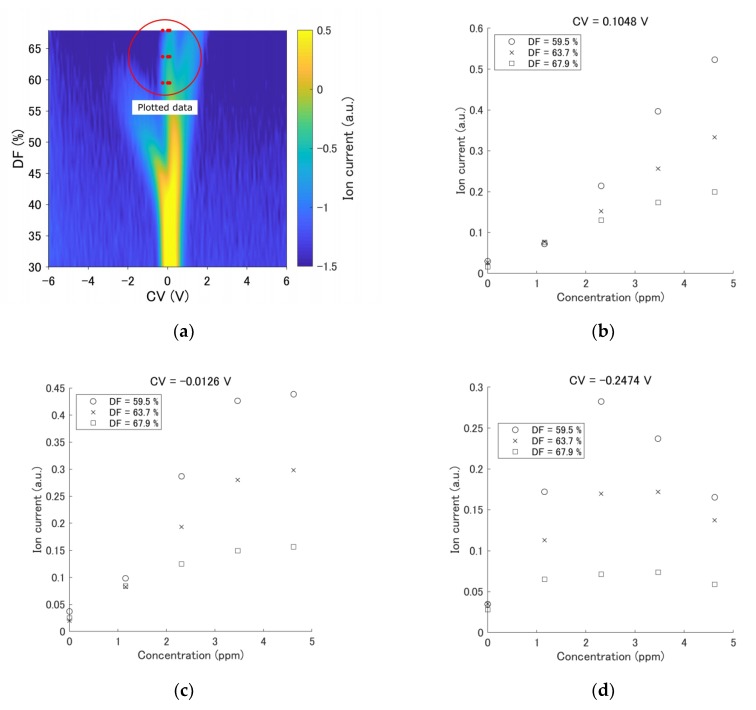

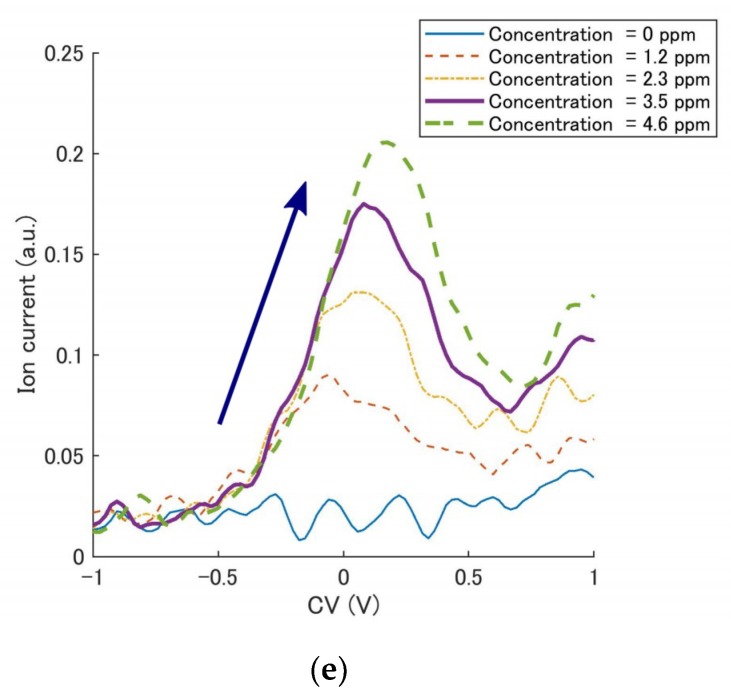
An example of a single component measurement: response to acetone as a function of concentration. Dispersion fields (DFs) of 59.5, 63.7, and 67.9% were selected. (**a**) The measurement result of 4.6 ppm acetone was obtained. The red circle indicates the portion of plotted data in the graphs. (**b**) The output at compensation voltage (CV) = 0.1048 V. (**c**) The output at CV = −0.0126 V. (**d**) The output at CV = −0.2474 V. (**e**) The output at DF = 67.9%, CV = −1 to 1 V.

FAIMS has nonlinearity characteristics, as shown in Figure 3b–e. For example, the output of a single component (acetone) with a concentration change is plotted in Figure 3b–d. The portion of plotted data in these figures is shown in Figure 3a. The change in the ion current (IC) depends on the CV and DF values. The output in Figure 3c was nonlinear in relation to the acetone concentration, whereas the output in Figure 3b was relatively linear in relation to that concentration. The IC exhibited a peak at the concentration change, as shown in Figure 3d. From a different point of view, these results indicate that the peak of the response was shifted due to the concentration change (see Figure 3e).

**Figure 4:**
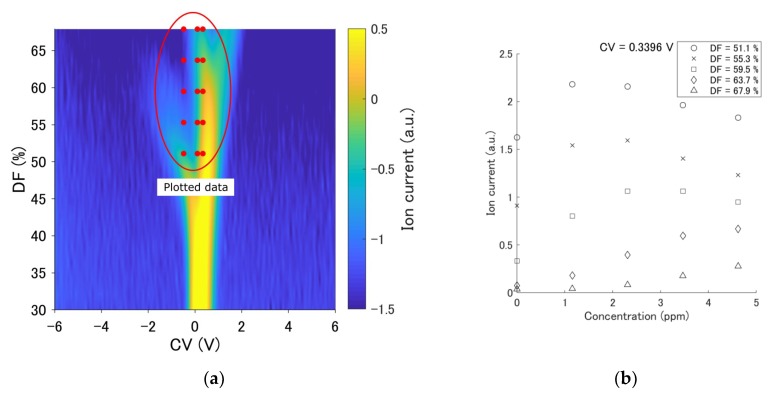

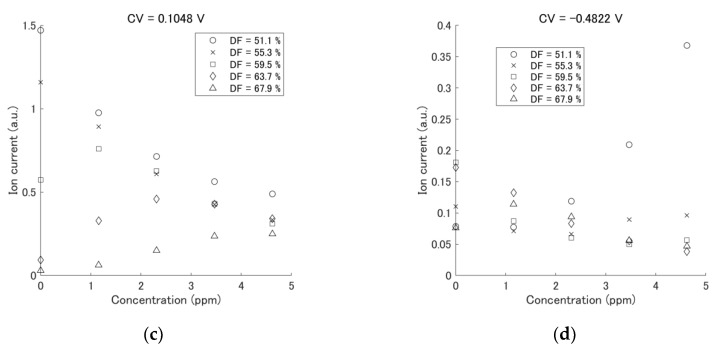
An example of a ternary gas mixture. The concentration of acetone was changed from 0 ppm to 4.6 ppm, and the concentrations of other gases were fixed at 6.1 ppm. DFs of 51.1, 55.3, 59.5, 63.7, and 67.9% were selected. (**a**) The measurement results indicated that the mixture included 4.6 ppm acetone, 6.1 ppm ethanol, and 6.1 ppm diethyl ether. The red circle indicates the portion of plotted data in the graphs. (**b**) The output at CV = 0.3396 V. (**c**) The output at CV = 0.1048 V. (**d**) The output at CV = −0.4822 V.

The measurement results using an example of a ternary gas mixture when the concentration of acetone mixed with ethanol (6.1 ppm) and diethyl ether (6.1 ppm) was changed are shown in Figure 4b–d. The change of the IC with the concentration change was more nonlinear than that observed for a single component. For instance, the results for DF = 51.1, 55.3, and 59.5% in Figure 4b reveal that the IC exhibited a peak; however, the output increased monotonically with the concentration, as shown in the results for DF = 63.7 and 67.9% in Figure 4b. The output decreased (DF = 51.1, 55.3%), or had a peak (DF = 59.5, 63.7%), or increased monotonically (DF = 67.9%), depending on the value of the DF, as shown in Figure 4c. Only one IC (DF = 51.1%) increased monotonically, whereas others were almost unchanged, as shown in the results in Figure 4d. A new approach is required for FAIMS quantification, because it is difficult to apply a simple regression method to FAIMS measurement due to its complicated nonlinear properties.

### 2.5. Gas Mixture Quantification

**Figure 5:**
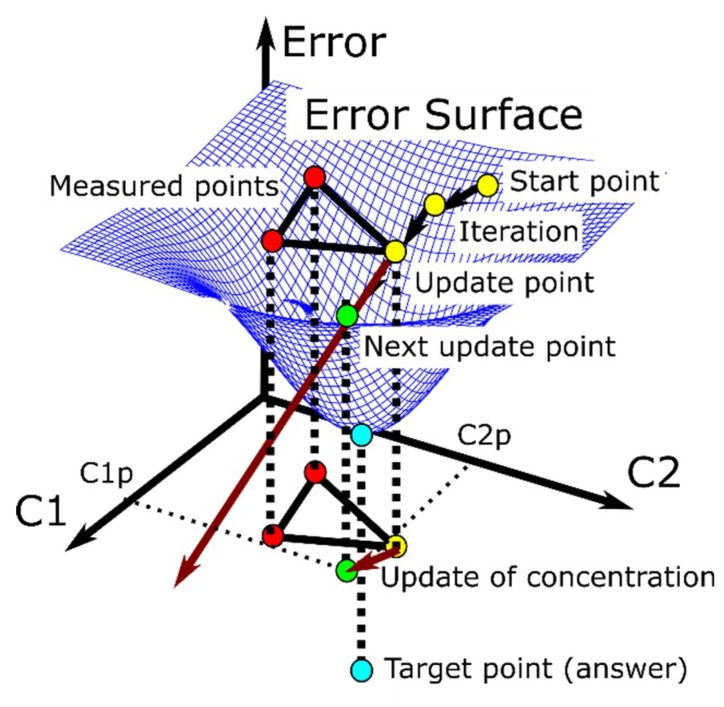
Gas mixture quantification for a binary gas mixture. The error surface is determined by the gas mixture composition and a target of gas mixture data. The gradient is calculated from three points, and the update point goes straight ahead in the direction of the maximum slope. This process is iterated until the update point reaches the target point, which has the minimum value in the error surface.

In our previous research, the gradient descent was used for gas mixture quantification [25]. Figure 5 explains the method for binary gas mixture quantification. The error E was calculated from the difference between each measurement ion current matrix $IC_{2}$ and target ion current matrix $IC_{1}$:

(2) $$E=\sum_{k=1}^{19}\sum_{l=1}^{512}\left|IC_{2kl}-IC_{1kl}\right|,$$

where each element indicates the intensity at the $k_{th}$ row and the $i_{th}$ column.

The concentrations at each measurement data point are known, whereas the concentrations at the target data points are unknown. It is difficult to obtain an actual error surface value because many measurement points are required. Gradient descent is used to search for the minimum error point in the error surface. Two measured points and one other measured point, which is called the update point, are initially selected (the red points and yellow point, respectively, in Figure 5). The gradient is calculated using those three points, and then the update point goes straight ahead in the direction of the maximum slope (*c_1p_*, *c_2p_* are the new concentrations in Figure 5). This process is iterated until the update point reaches the target point, which has the minimum value in the error surface (the blue point in Figure 5).

The number of dimensions can be extended to *m*. The error hypersurface is also defined as

(3) $$\;E=f\left(c_{p}\right)=f\left(c_{1p},c_{2p},\ldots ,c_{mp}\right),$$

where $c_{p}$ is the concentration vector at the update point. The function f can be expanded around update point:

(4) $$\;E-E_{p}={\frac{\partial f}{\partial c_{1}}|}_{c_{1}=c_{1p}}\left(c_{1}-c_{1p}\right)+,\ldots ,+{\frac{\partial f}{\partial c_{i}}|}_{c_{i}=c_{ip}}\left(c_{i}-c_{ip}\right)+,\ldots ,+{\frac{\partial f}{\partial c_{m}}|}_{c_{m}=c_{mp}}\left(c_{m}-c_{mp}\right),$$

where$\;E_{p}$ is E at the update point and$\;c_{ip}$ is the concentration of the *i_th_* component at the update point. If ΔI, ΔC, and ∂f/∂c are defined as

(5) $$\;\Delta I=\left[\begin{array}{c} E_{1}-E_{p}\\ \vdots \\ E_{m}-E_{p} \end{array}\right],\;\;\Delta C=\left[\begin{array}{ccc} c_{11}-c_{1p} & \cdots & c_{m1}-c_{mp}\\ \vdots & c_{ij}-c_{ip} & \vdots \\ c_{1m}-c_{1p} & \cdots & c_{mm}-c_{mp} \end{array}\right],\;\frac{\partial f}{\partial c}={\left[\frac{\partial f}{\partial c_{1}},\cdots ,\frac{\partial f}{\partial c_{m}}\right]}^{T},$$

where m is the number of data points and $c_{ij}$ is the concentration of the $i_{th}$ component of the $j_{th}$ data point. The equation

(6) $$\;\left[\Delta I\right]=\left[\Delta C\right]\left[\frac{\partial f}{\partial c}\right]$$

is obtained from the Equations (4) and (5). The following equation is obtained by solving (4)

(7) $$\;\left[\frac{\partial f}{\partial c}\right]={\left[\Delta C\right]}^{-1}\left[\Delta I\right].$$

The next update point is obtained using (6)

(8) $$\;c_{next\_p}=c_{p}-\varepsilon \left[\frac{\partial f}{\partial c}\right],$$

where ε is the empirically determined learning rate. It decides the speed of convergence.

### 2.6. Automatic Adjustment of the Learning Rate 

The learning rate ε is what determines the next step size. A constant value was used in previous research; however, it is very difficult to find a suitable learning rate, because it greatly depends on the characteristics of the measured data. The AdaGrad algorithm was adopted for this purpose [26]. The implementation of the AdaGrad algorithm requires that the calculated gradient history be recorded for the gradient descent calculation. Each learning rate per component is divided by the square root of the summation of the squared values in the gradient history per component [27]:

(9) $$\varepsilon_{i,M}=\frac{\varepsilon_{init\_i}}{\sqrt{\sum_{k=1}^{M}w_{i,k}^{2}}},$$

where $\varepsilon_{i,M}$ is the $M_{th}$ learning rate of the $i_{th}$ component, $w_{i,k}$ is the $k_{th}$ gradient of the $i_{th}$ component, and $\varepsilon_{init\_i}$ is the initial learning rate of the $i_{th}$ component.

## 3. Results and Discussion

**Figure 6:**
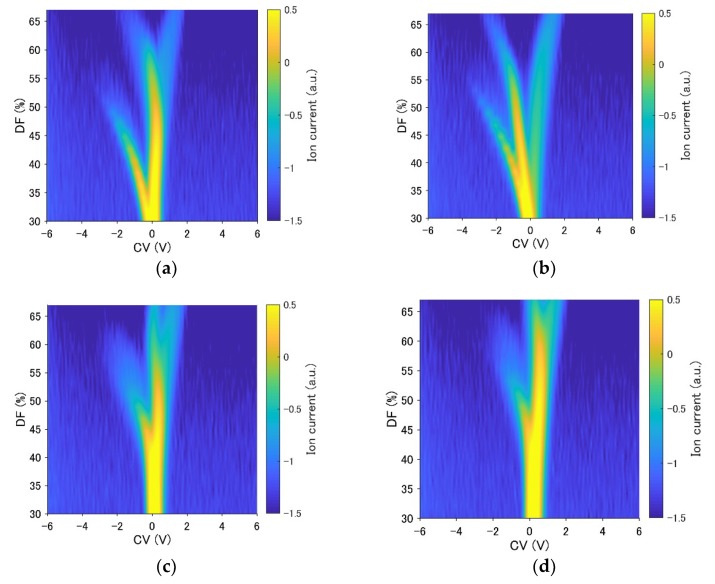
Part of the measurement results of pure gases and a ternary gas mixture. (**a**) Pure diethyl ether (6.1 ppm). (**b**) Pure ethanol (6.1 ppm). (**c**) Pure acetone (4.6 ppm). (**d**) The ternary mixture of acetone (4.6 ppm), ethanol (6.1 ppm), and diethyl ether (6.1 ppm).

**Figure 7:**
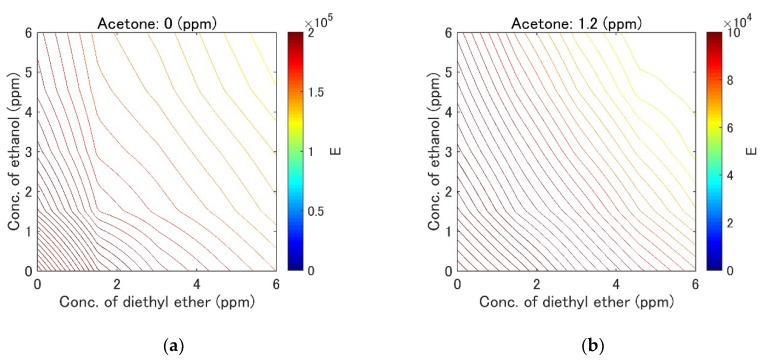

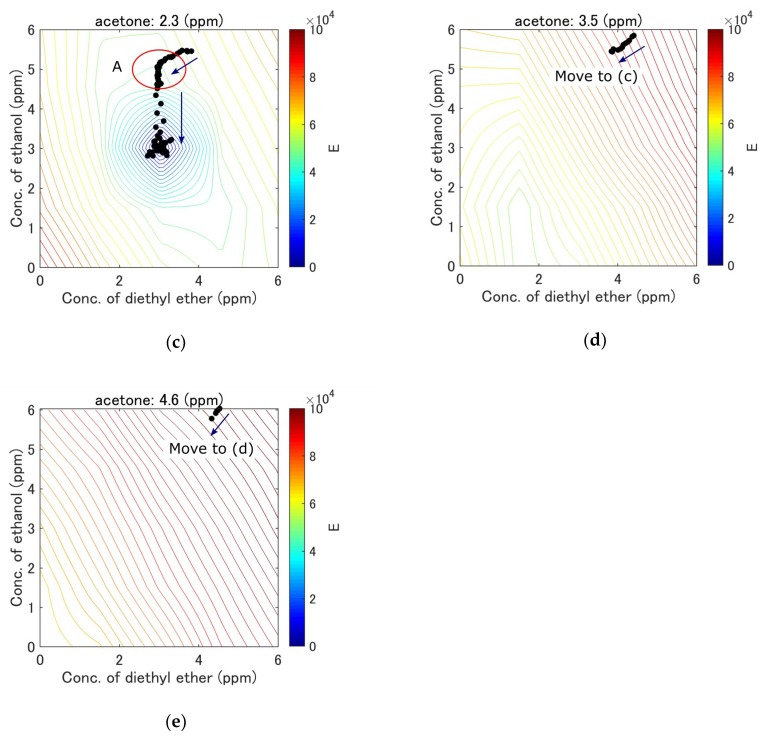
Contour maps of the error hypersurfaces obtained from all the measurement data. The concentration of acetone was fixed to prepare the contour maps. (**a**) 0 ppm, (**b**) 1.2 ppm, (**c**) 2.3 ppm, (**d**) 3.5 ppm, (**e**) 4.6 ppm. Black points mean the update point history.

**Figure 8:**
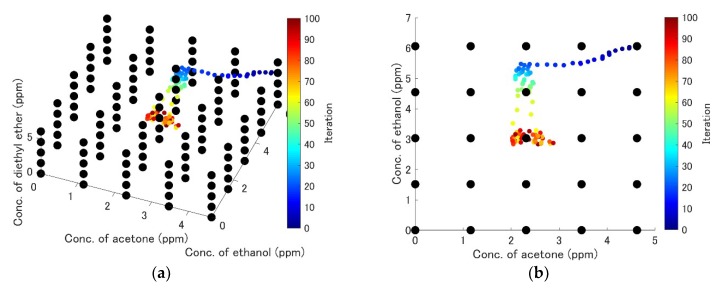

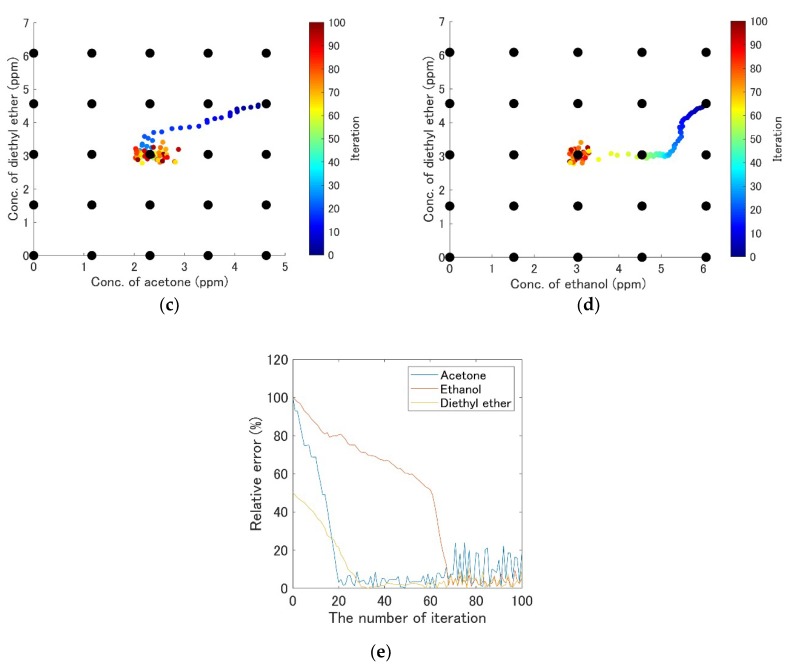
The result of the determination of each gas concentration using the gradient descent method. The error hypersurface calculated from all measurement data was used for the gradient descent. The initial measurement points were acetone (4.6 ppm), ethanol (6.1 ppm), and diethyl ether (4.6 ppm). Acetone (2.3 ppm), ethanol (3.0 ppm), and diethyl ether (3.0 ppm) were selected for the target data. ε was $1.0\;\times 10^{-5}$. (**a**) 3D view. (**b**) Acetone–ethanol plane. (**c**) Acetone–diethyl ether plane. (**d**) Ethanol–diethyl ether plane. (**e**) The history of relative errors.

**Figure 9:**
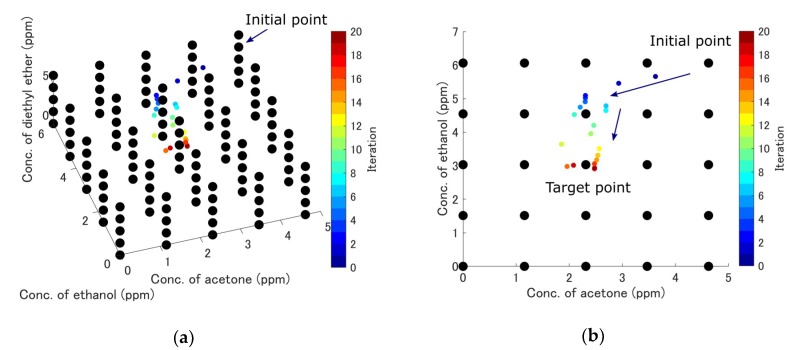

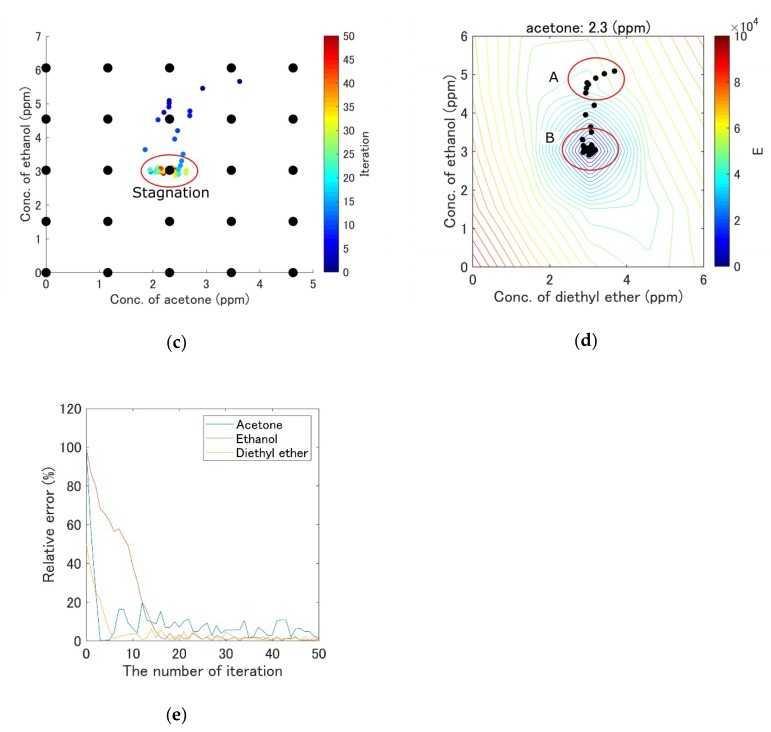
The results of the learning rate auto adjustment. (**a**) 3D view. (**b**) Acetone–ethanol plane. (**c**) Acetone–ethanol plane when the number of iteration increases. (**d**) The update history and the contour map of the error hypersurfaces with fixed concentration of acetone (2.3 ppm). (**e**) The history of relative errors. The initial values of $\varepsilon_{init}$ were 1 (acetone), 0.4 (ethanol), and 0.4 (diethyl ether).

A subset of the measurements, including the measurements of pure component gases and a typical gas mixture, is shown in Figure 6a–d. Contour maps of the error hypersurfaces obtained from all the measurement data are shown in Figure 7a–e. The mixture of acetone (2.3 ppm), ethanol (3.0 ppm), and diethyl ether (3.0 ppm) was selected as the target mixture. Linear interpolation was applied to obtain the continuous error hypersurface. The trajectory of the exploration is shown in Figure 8a–d. The initial position (acetone: 4.6 ppm, ethanol: 6.1 ppm, diethyl ether: 4.6 ppm) and the target position (acetone: 2.3 ppm, ethanol: 3.0 ppm, diethyl ether: 3.0 ppm) were selected. ε was kept constant at $1.0\;\times 10^{-5}$. The update history was also plotted as black dots in Figure 7c–e. Relative errors were calculated using 30 points of update history after reaching the target point for accuracy evaluation. The mean relative errors were 4.8%, 0.63%, 0.67%, and their standard deviations were 8.1%, 2.2%, 2.93%. The history of relative error is shown in Figure 8e. The results of quantification with the automatic adjustment of the learning rate is shown in Figure 9a–c. The initial values of ε were set to 1, 0.4, and 0.4 for acetone, ethanol, and diethyl ether, respectively. Figure 9a–b show the convergence of the quantification within 20 iterations. The update point was still found to stagnate near the target point if the number of iterations was increased (Figure 9c). The update history and the contour map of the error hypersurfaces with a fixed concentration of acetone (2.3 ppm) are shown in Figure 9d. Relative errors were also calculated, the mean relative errors were 0.2%, 0.07%, 0.01%, and the standard deviations were 3.0%, 1.0%, 1.4%, respectively. The history of relative error is also shown in Figure 9e. The differences of variances between the method of constant learning rate and adaptive learning rate were investigated by the Ansari-Bradley test. Thirty points obtained from the two methods were also used. The median values of two sample data were almost the same (the differences were 0.0102 ppm, 0.0289 ppm, 0.0024 ppm for three components). The significance level was 5%, and the p values were 0.0143, $4.9875\times 10^{-5}$, 0.0044, respectively. Ansari-Bradley test shows that there are differences between the two methods for all three components.

If the error hypersurface is flat, this means that FAIMS cannot distinguish among gas mixtures with different concentrations. Although Figure 6a–d show that there is a difference among the measurement results of all gas components and the mixture of these components, the change did not appear to be significant when the data were visualized. However, Figure 7a–e indicate that FAIMS can distinguish among those gas mixtures with different concentrations, since the error hypersurface exhibited a gradient toward the target point.

It is important to obtain the value of the error hypersurface, because it greatly influences the performance of the proposed quantification method. Equation (2) was used to determine the error hypersurface. This method is useful despite its simplicity, because the error hypersurface was as expected. The cost function near the center is low, whereas its value away from the center becomes high, demonstrating an appropriate slope. The quality of the error hypersurface could be further improved if a more sophisticated method were used.

The convergence to the target point was confirmed using the gradient descent method. When the number of iterations increased, the update point stagnated near the target point (Figure 8a–d). This means that the update point converged to the target point and the quantification was successful. The update history in Figure 7c–e shows that the update point moved in the direction across the contour of the error surface, before finally stagnating at the portion with low error. The update point movement was slow near portion A in Figure 7c because the gradient around that area was low. This is because the influence of the ethanol concentration change was smaller than those of other factors.

The results of Figure 9a–c show that the number of iterations could be reduced to 20, much less than that required in Figure 8a–d. Quantification can be done in real time if the number of iterations is small [25]. The learning rate of component 1 ranged from $6.2756\times 10^{-5}$ to $8.0709\times 10^{-6}$, that of component 2 ranged from $1.2821\times 10^{-4}$ to $5.8514\times 10^{-6}$, and that of component 3 ranged from $1.1168\times 10^{-4}$ to $6.5663\times 10^{-6}$. The remarkable change of the learning rate improved the convergence performance drastically. This is because the large step size helped to avoid slowing down near the position with low gradient (the portion A in Figure 9d), and decreasing the learning rate helped to avoid instability near the position with a high gradient (the portion B in Figure 9d). The method also improved the accuracy, because the mean values of relative error and variances thereof were decreased. The difference of variances between the two methods was statistically significant. The initial learning rate in this study was chosen to balance the number of iterations with accuracy. Accuracy can increase if a smaller learning rate is selected; however, this requires a long time for the convergence.

We cannot compare FAIMS with other methods because, to our knowledge, this is the first time that FAIMS has been employed for mixture quantification. Other groups generally used FAIMS for classification; however, it has been used for mixture quantification. This is challenging task, because quantification is more difficult than identification. A previous paper reported that a QCM sensor array was able to quantify up to eight components of a gas mixture [28]. The number of components in the gas mixture should be increased and real-time quantification should be realized for the potential application of this method to odor recorders and reproducers.

## 4. Conclusions

One hundred and twenty-five mixture compositions of a ternary gas mixture were measured, and the gradient descent was used to quantify their concentrations. Its convergence was successfully achieved using the proposed method, though a nonlinear behavior of FAIMS was observed. The learning rate was adaptively adjusted to reduce the number of iterations, and it was found that the number of iterations could be drastically decreased. This result suggests the feasibility of odor quantification in real time. Halitosis-substance sensing is promising for the application of this technology, because FAIMS can quantify gases such as volatile sulfur compounds (VSCs) [29] without a preconcentrator under high-water conditions. This study will also be extended to a gas mixture with more constituents and real-time quantification for future applications.

## References

[1] Yamanaka T., Matsumoto R., Nakamoto T. (2002). Study of odor blender using solenoid valves controlled by delta–sigma modulation method for odor recorder. Sens. Actuators B Chem..

[2] Nakamoto T., Nakamoto T. (2016). Olfactory Display and Odor Recorder. Essentials of Machine Olfaction and Taste.

[3] Choh N., Wyszynsky B., Takushima H., Nitikarn N., Kinoshita M., Nakamoto T. (2008). Demonstration of Interactive Teleolfaction with Movie. Proceedings of the 2008 International Conference on Advances in Computer Entertainment Technology.

[4] Nakamoto T., Otaguro S., Kinoshita M., Nagahama M., Ohinishi K., Ishida T. (2008). Cooking up an Interactive Olfactory Game Display. IEEE Comput. Graph. Appl..

[5] Eiceman G.A., Karpas Z., Hill H.H. (2016). Ion Mobility Spectrometry.

[6] Keller T., Miki A., Regenscheit P., Dirnhofer R., Schneider A., Tsuchihashi H. (1998). Detection of designer drugs in human hair by ion mobility spectrometry (IMS). Forensic Sci. Int..

[7] Ewing R.G., Atkinson D.A., Eiceman G.A., Ewing G.J. (2001). A critical review of ion mobility spectrometry for the detection of explosives and explosive related compounds. Talanta.

[8] Shvartsburg A.A. (2009). Differential Ion Mobility Spectrometry: Nonlinear Ion Transport and Fundamentals of FAIMS.

[9] Shvartsburg A.A. (2009). Conceptual Implementation of Differential IMS and Separation Properties of FAIMS. Differential Ion Mobility Spectrometry: Nonlinear Ion Transport and Fundamentals of FAIMS.

[10] Rutolo M., Covington J.A., Clarkson J., Iliescu D. (2014). Detection of Potato Storage Disease via Gas Analysis: A Pilot Study Using Field Asymmetric Ion Mobility Spectrometry. Sensors.

[11] Sinha R., Khot L.R., Schroeder B.K., Sankaran S. (2018). FAIMS based volatile fingerprinting for real-time postharvest storage infections detection in stored potatoes and onions. Postharvest Biol. Technol..

[12] Covington J.A., der Schee M.V., Edge A.S.L., Boyle B., Savage R.S., Arasaradnam R.P. (2015). The application of FAIMS gas analysis in medical diagnostics. Analyst.

[13] Covington J.A., Wedlake L., Andreyev J., Ouaret N., Thomas M.G., Nwokolo C.U., Bardhan K.D., Arasaradnam R.P. (2012). The Detection of Patients at Risk of Gastrointestinal Toxicity during Pelvic Radiotherapy by Electronic Nose and FAIMS: A Pilot Study. Sensors.

[14] Sahota A.S., Gowda R., Arasaradnam R.P., Daulton E., Savage R.S., Skinner J.R., Adams E., Ward S.A., Covington J.A. (2016). A simple breath test for tuberculosis using ion mobility: A pilot study. Tuberculosis.

[15] Arasaradnam R.P., Wicaksono A., O’Brien H., Kocher H.M., Covington J.A., Crnogorac-Jurcevic T. (2018). Noninvasive Diagnosis of Pancreatic Cancer through Detection of Volatile Organic Compounds in Urine. Gastroenterology.

[16] Plat V.D., van Gaal N., Covington J.A., Neal M., de Meij T.G.J., van der Peet D.L., Zonderhuis B., Kazemier G., de Boer N.K.H., Daams F. (2019). Non-Invasive Detection of Anastomotic Leakage Following Esophageal and Pancreatic Surgery by Urinary Analysis. Dig. Surg..

[17] Pfammatter S., Bonneil E., McManus F.P., Thibault P. (2019). Accurate Quantitative Proteomic Analyses Using Metabolic Labeling and High Field Asymmetric Waveform Ion Mobility Spectrometry (FAIMS). J. Proteome Res..

[18] Kontunen A., Karjalainen M., Lekkala J., Roine A., Oksala N. (2018). Tissue Identification in a Porcine Model by Differential Ion Mobility Spectrometry Analysis of Surgical Smoke. Ann. Biomed. Eng..

[19] Sutinen M., Kontunen A., Karjalainen M., Kiiski J., Hannus J., Tolonen T., Roine A., Oksala N. (2019). Identification of breast tumors from diathermy smoke by differential ion mobility spectrometry. Eur. J. Surg. Oncol..

[20] Surakka V. (2016). From electrical scent analysis to digital scent production. Proceedings of the Journal of Digital Olfaction Society (JDOS).

[21] Li J., Gutierrez-Osuna R., Hodges R.D., Luckey G., Crowell J., Schiffman S.S., Nagle H.T. (2016). Using Field Asymmetric Ion Mobility Spectrometry for Odor Assessment of Automobile Interior Components. IEEE Sens. J..

[22] Wilks A., Hart M., Koehl A., Somerville J., Boyle B., Ruiz-Alonso D. (2012). Characterization of a miniature, ultra-high-field, ion mobility spectrometer. Int. J. Ion Mobil. Spectrom..

[23] Eiceman G.A., Karpas Z., Hill H.H. (2016). Gas Chromatography. Ion Mobility Spectrometry.

[24] Hunter E.P.L., Lias S.G. (1998). Evaluated Gas Phase Basicities and Proton Affinities of Molecules: An Update. J. Phys. Chem. Ref. Data.

[25] Nakamoto T., Ustumi S., Yamashita N., Moriizumi T., Sonoda Y. (1994). Active gas/odor sensing system using automatically controlled gas blender and numerical optimization technique. Sens. Actuators B Chem..

[26] Duchi J., Hazan E., Singer Y. (2011). Adaptive Subgradient Methods for Online Learning and Stochastic Optimization. J. Mach. Learn. Res..

[27] Dean J., Corrado G., Monga R., Chen K., Devin M., Mao M., Ranzato M.A., Senior A., Tucker P., Yang K., Pereira F., Burges C.J.C., Bottou L., Weinberger K.Q. (2012). Large Scale Distributed Deep Networks. Advances in Neural Information Processing Systems 25.

[28] Yamanaka T., Matsumoto R., Nakamoto T. (2003). Fundamental study of odor recorder for multicomponent odor using recipe exploration method based on singular value decomposition. IEEE Sens. J..

[29] Ito J., Nakamoto T., Uematsu H. (2004). Discrimination of halitosis substance using QCM sensor array and a preconcentrator. Sens. Actuators B Chem..

